# Host Immunity Escalates the Evolution of Parasite Virulence

**DOI:** 10.1371/journal.pbio.0020251

**Published:** 2004-06-22

Strictly defined, evolution is a change in the gene pool, or total set of genes, of a given population over time. Genetic changes that increase the fitness of an organism—that is, increase survival or fertility—are more likely to be retained, through natural selection, and passed on to succeeding generations. In the classic case of Darwin's finches, different ecological niches exerted different selective pressures on an original population, and resulted in 14 different species, each sporting a beak uniquely adapted to harvesting particular available food sources.

When it comes to microbial evolution, an ecological niche often takes the form of a host. If the microbe is a pathogen, its presence might trigger strong selective pressure from the host's immune system, precipitating an evolutionary two-step between microbe and host. Hosts with strong immune defenses can typically tolerate relatively virulent pests: conversely, ill-defended hosts die, which is bad news for the parasite. When the myxoma virus first infected a population of European rabbits in Australia in 1950, the virus was particularly lethal. Over time, less virulent strains were selected for—killing off your habitat would be an unsustainable fitness cost by most standards—and the rabbits developed resistance.

In keeping with evolutionary theory, host immunity should affect the evolution of parasite virulence. Though theory predicts that immunity could potentially heighten virulence, there's no evidence that this is true. Being able to predict how natural selection will act on, and thus shape, virulence is vital for developing effective public health policies—and desperately needed vaccines—to deal with the ever growing roster of rapidly evolving pathogenic threats.[Fig pbio-0020251-g001]

**Figure pbio-0020251-g001:**
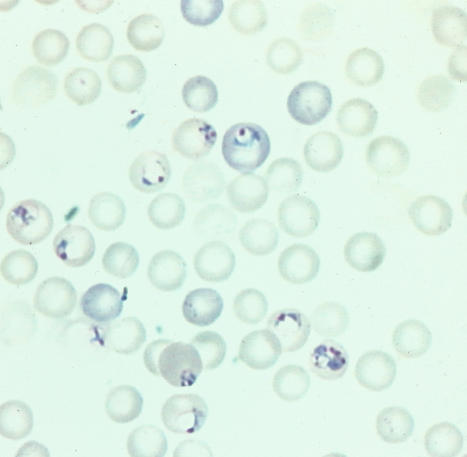
Malaria infecting red blood cells

To investigate whether immune system defenses escalate pathogen virulence, Margaret Mackinnon and Andrew Read studied the malarial parasite Plasmodium in the mouse. Mackinnon and Read first directly injected two groups of mice with infectious parasites: “immunized” mice, which had been exposed to Plasmodium and then treated with the antimalarial drug mefloquine, and “naïve” mice, which had not. Parasites were serially transferred twenty times via a syringe from one mouse host to another. The virulence and infectiousness of the respective strains were evaluated by introducing the strains into another set of immunized and naïve mice.

As theoretically predicted, parasites evolved in the immunized mice were indeed more virulent than parasites evolved in the naïve mice. But what if the parasites were first transmitted through their natural vector, the mosquito, rather than through a syringe? Would they be as virulent? Interestingly, infection was not as severe after mosquito transmission. But parasites evolved in the immunized mice retained a higher level of virulence than those evolved in the naïve mice. This means that immunity accelerates the evolution of virulence in malaria, even after mosquito transmission, making them more dangerous to nonimmunized hosts.

How does immune selection create more virulent pathogens? One possibility is that even though many parasites die in immunized hosts, those that “win the race to the syringe”—or the mosquito—are likely genetically equipped to stay ahead of advancing immune system defenses.

It's not entirely clear why selection would favor more virulent parasites, but since the virulent strains showed no problems transmitting infection to new hosts, it's likely that such strains would spread throughout an immunized population. While mosquito transmission likely plays a significant role in virulence evolution—it clearly reduced virulence here—the molecular mechanics of this effect are also mostly speculative at this point. Many questions remain, but these results make a strong case that vaccine development aimed at protecting individuals against infectious pathogens would do well to consider the evolutionary implications, or increased pathogen virulence could be an unintended consequence.

